# Crucial role of the transcription factors family activator protein 2 in cancer: current clue and views

**DOI:** 10.1186/s12967-023-04189-1

**Published:** 2023-06-08

**Authors:** Chen Jin, Yuxiao Luo, Zhu Liang, Xi Li, Damian Kołat, Linyong Zhao, Weixi Xiong

**Affiliations:** 1 Department of Cardiothoracic Surgery, The First Affiliated Hospital of Chongqing Medical University, Chongqing, China; 2 University Medical Center Göttingen, University of Göttingen, Göttingen, Germany; 3 Target Discovery Institute, Centre for Medicines Discovery, Nuffield Department of Medicine, University of Oxford, Chinese Academy for Medical Sciences Oxford Institute, Oxford, UK; 4 Department of Urology, Churchill Hospital, Oxford University Hospitals NHS Foundation, Oxford, UK; 5 Department of Experimental Surgery, Medical University of Lodz, Lodz, Poland; 6 Department of General Surgery & Laboratory of Gastric Cancer, State Key Laboratory of Biotherapy/Collaborative Innovation Center of Biotherapy and Cancer Center, West China Hospital, Sichuan University, Chengdu, China; 7 Gastric Cancer Center, West China Hospital, Sichuan University, Chengdu, China; 8 Department of Neurology, West China Hospital, Sichuan University, Chengdu, China; 9 Institute of Brain Science and Brain-Inspired Technology, West China Hospital, Sichuan University, Chengdu, China; 10 Department of Liver Surgery, West China Hospital, Sichuan University, Chengdu, China

**Keywords:** Activator protein 2, Transcription factor, Cancer stemness, Epithelial-mesenchymal transition, Tumor microenvironment, Ferroptosis

## Abstract

The transcription factor family activator protein 2 (TFAP2) is vital for regulating both embryonic and oncogenic development. The TFAP2 family consists of five DNA-binding proteins, including TFAP2A, TFAP2B, TFAP2C, TFAP2D and TFAP2E. The importance of TFAP2 in tumor biology is becoming more widely recognized. While TFAP2D is not well studied, here, we mainly focus on the other four TFAP2 members. As a transcription factor, TFAP2 regulates the downstream targets directly by binding to their regulatory region. In addition, the regulation of downstream targets by epigenetic modification, posttranslational regulation, and interaction with noncoding RNA have also been identified. According to the pathways in which the downstream targets are involved in, the regulatory effects of TFAP2 on tumorigenesis are generally summarized as follows: stemness and EMT, interaction between TFAP2 and tumor microenvironment, cell cycle and DNA damage repair, ER- and ERBB2-related signaling pathway, ferroptosis and therapeutic response. Moreover, the factors that affect TFAP2 expression in oncogenesis are also summarized. Here, we review and discuss the most recent studies on TFAP2 and its effects on carcinogenesis and regulatory mechanisms.

**Supplementary Information:**

The online version contains supplementary material available at 10.1186/s12967-023-04189-1.

## Background

The transcription factor family activator protein 2 (TFAP2) has been found to be critical for controlling gene expression during embryonic development and differentiation. Recently, its indispensable role in carcinogenesis has been increasingly discussed. TFAP2 comprises five members, namely, TFAP2A, TFAP2B, TFAP2C, TFAP2D and TFAP2E. With the exception of TFAP2D, which has distinct sequence specificity, the other four TFAP2 members share highly conserved domains, such as a basic domain, a helix-span-helix motif and a proline- and glutamine-rich transactivation domain (a PY motif within it). Of note, the carboxyl terminus contains the helix-span-helix motif, which is crucial for DNA binding and dimerization. TFAP2 tends to bind to GC-rich cis-regulatory elements when interacting with target genes [1].

As previously summarized in the literature, functions of TFAP2A, TFAP2B and TFAP2C have been discovered in the early development of neural crest cells, the peripheral nervous system, facial and limb mesenchyme, various epithelia and the extraembryonic trophectoderm, while TFAP2D is restricted to the developing heart, retina and central nervous system. TFAP2E is found in cells of the olfactory bulb [1–4].

In oncogenesis, early investigations revealed the correlation between TFAP2 expression level and tumor malignancy and patient prognosis. For instance, in gastric adenocarcinoma, reduced TFAP2A expression was associated with advanced tumor stage and poor prognosis [5]. Low nuclear expression of TFAP2A in ovarian cancer is related to poor prognosis [6]. TFAP2B executes an anti-apoptotic function in alveolar rhabdomyosarcoma [7]. TFAP2C expression is increased in advanced ovarian carcinoma [8]. Upregulated TFAP2D is correlated with enhanced malignancy and poor prognosis [9]. The expression of TFAP2E is associated with chemoresistance [10]. Both concordant and contradictory results regarding the expression patterns and functions of TFAP2 members have been observed; thus, a thorough understanding of the role of TFAP2 in carcinogenesis is urgently needed. Due to limited reports concerning the function of TFAP2D in cancer, we focused on describing the other four TFAP2 members.

To comprehensively summarize the role of TFAP2 in cancers, we have retrieved literature as much as possible by searching MEDLINE and Embase databases. To avoid missing any information due to the synonyms or aliases for ”Transcription Factor AP 2” and “Neoplasm”, we searched these two keywords by MeSH search in the MEDLINE database (up to April 2023) and by Emtree search in the Embase database (up to April 2023). Afterward, we made a thorough literature screening by reading abstracts and full texts in line with the PRISMA flow [11]. Finally, we summarized experimentally validated results to categorize TFAP2-regulated biological processes in cancers comprehensively. The screening flow diagram is shown in (Additional file 1: Figure S1).

## Regulatory modes of TFAP2 on downstream targets

The TFAP2 protein primarily exerts its function at the transcriptional level by directly binding to the promoters of target genes. Therefore, the collective effects of TFAP2 on tumors depend on the downstream targets (tumor promotion or suppressive gene). In addition, distinct binding sites on the same promoter can also result in varied regulation outcomes. In pancreatic adenocarcinoma, TFAP2A interacts with the mucin 4 (*MUC4*) promoter to suppress MUC4 expression, which prevents cell proliferation and invasion [12].

Epigenetically, TFAP2 alters chromatin conformation to change its accessibility to the regulatory region. For example, TFAP2A increases enhancer of zeste 2 polycomb repressive complex 2 subunit (*EZH2*) expression by perturbing the activity of the nucleosome remodeling and deacetylase (NuRD) complex to enhance chromatin accessibility to the *EZH2* promoter region, which facilitates transcriptional activation [13]. Furthermore, in breast cancer, the interaction between TFAP2C and metastasis-associated protein 1 (MTA1), a member of the nucleosome remodeling and HDAC complex, induces estrogen receptor (ER) expression via epigenetic chromatin modification [14].

In addition, the TFAP2 protein occupies the regulatory region of target genes competitively with other coregulators. Competitive binding between TFAP2 and specificity protein 1 (SP1) has been shown to be important in the regulation of other target genes. In metastatic melanoma cells, decreased TFAP2A changes the binding ratio of TFAP2A/SP1 on the protease-activated receptor-1 (*PAR1*) promoter, which results in overexpression of PAR1 and promotes tumor oncogenesis and malignancy [15]. Moreover, reduced binding of TFAP2A to the peroxisome proliferator-activated receptors β/δ (*PPARB/D*) promoter leads to increased PPARβ/δ expression, in which TFAP2A and SP1 compete for occupancy on its promoter. Collectively, tumor proliferation occurs in non-small cell lung cancer (NSCLC) [16]. Indirectly, TFAP2A can activate the target genes of RNA polymerase III (Pol III) by transcriptionally upregulating Pol III subunits, such as BRF1 and GTF3C2 [17].

At the posttranslational level, the interaction between TFAP2 and other proteins that form a complex influences downstream targets or participates in subcellular translocation. Interaction between SRY-box transcription factor 8 (SOX8) and TFAP2A increases Golgi phosphoprotein 3 (GOLPH3) promoter activity and tumor growth of tongue squamous cell carcinoma [18]. WW Domain Containing Oxidoreductase (WWOX) protein acts as a tumor suppressor by interacting with TFAP2A and 2C. Previous studies discovered that both WWOX/TFAP2A and WWOX/TFAP2C display antitumor functions in intermediate grade BLCA. However, in high-grade (3 and 4) BLCA, different WWOX combinations showed distinct outcomes. WWOX/TFAP2A demonstrates anti-tumor functions, while WWOX/TFAP2C still promotes oncogenesis [19–22].

Tripartite motif-containing 37 (TRIM37) facilitates TFAP2C-mediated transcriptional activity on ERα in breast cancer. TRIM37 induces K63 chain-linked ubiquitination of TFAP2C, which facilitates the translocation of TFAP2C to the nucleus from the cytoplasm [23]. Furthermore, TFAP2C interacts with the signal transducer and activator of transcription 3 (STAT3) to form the TFAP2C/STAT3 complex and thus perturbs STAT3 phosphorylation, which prevents phosphorylated STAT3 from entering the nucleus to trigger fucosyltransferase 8 (*FUT8*) transcription [24]. FUT8 has been linked to tumor proliferation, metastasis, stemness and chemotherapy resistance. In addition, a small ubiquitin-like modifier (SUMOylating) controls the regulation activity of TFAP2, which will be discussed in the following section. Protein kinase A-mediated phosphorylation and redox regulation are observed in other biological processes [25, 26], but limited research has discussed these regulations’ effects on TFAP2 activity in tumors.

**Fig. 1:**
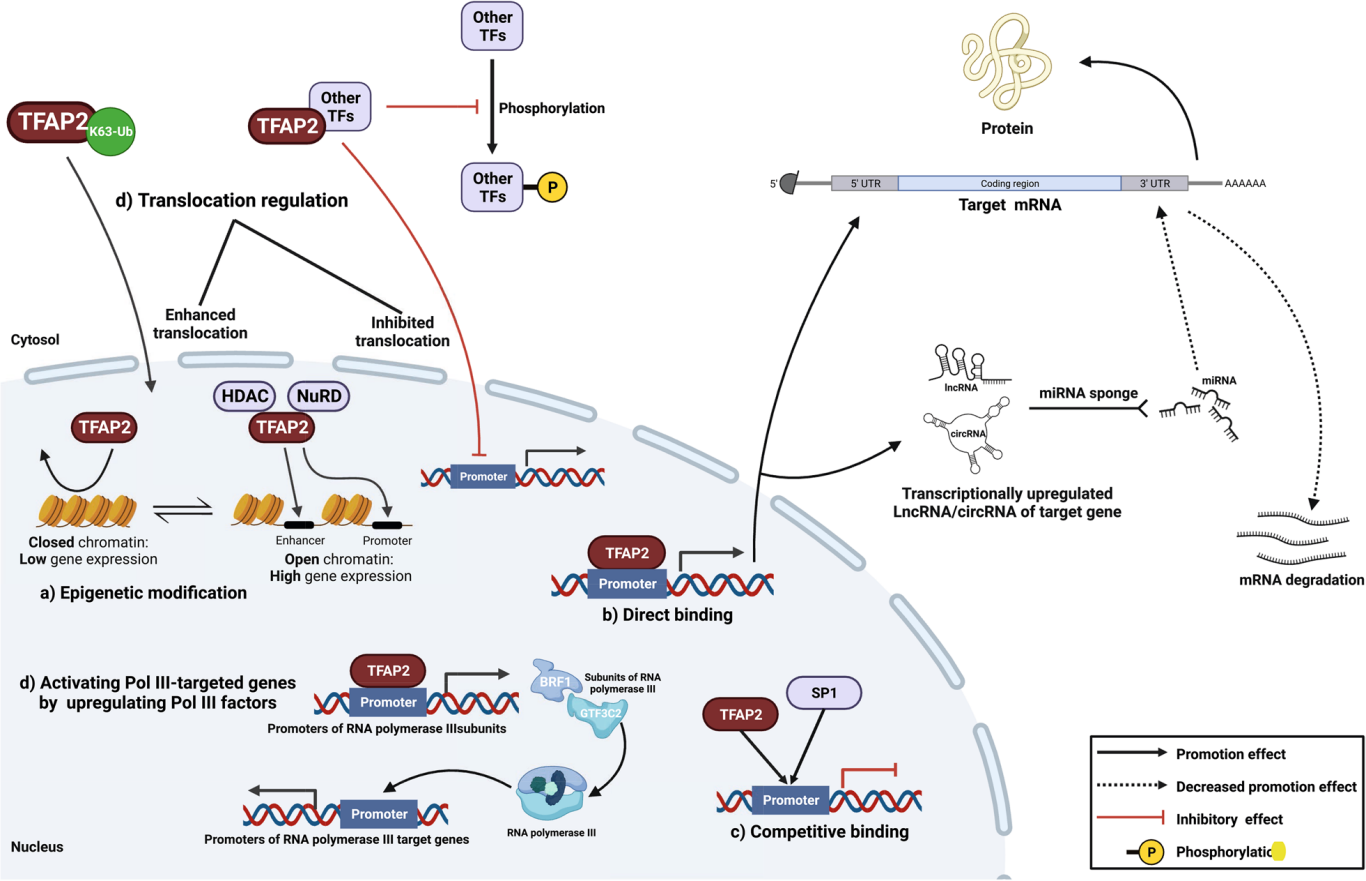
Regulatory modes of TFAP2 on downstream targets Regulatory modes of TFAP2 on downstream targets, **a** epigenetic modification on chromatin conformation to alter its accessibility to the regulatory region; **b** directly or **c** competitive binding on regulation region of the target gene and **d** posttranslational regulation, such as translocation shifting. Only validated direct protein–protein interaction and DNA/RNA–protein interaction are shown. (Created with BioRender.com)

The interaction between TFAP2 and noncoding RNA was also found to regulate the target gene. TFAP2C facilitates the proliferation, migration, and invasion of colorectal cancer by triggering the PI3K (phosphoinositide 3-kinases) /AKT (AKT serine/threonine kinase, also known as protein kinase B, PKB) signaling pathway. TFAP2C enhances circular RNA of IL4R (circIL4R) expression, which competitively interacts with microRNA 761 (miR-761) to promote the expression of tripartite motif-containing 29 (TRIM29). Subsequently, TRIM29 induces proteasome-mediated degradation of the PH domain and leucine-rich repeat protein phosphatase 1 (PHLPP1) and activates the PI3K/AKT signaling pathway [27]. A graphical schematic of the regulatory modes is summarized in Figure 1.

## The functions of TFAP2 in different aspects of tumor biology

According to our literature review and summary, the functions of TFAP2 are primarily involved in the following aspects: stemness and EMT, interaction with the tumor microenvironment, cell cycle and DNA damage repair, estrogen receptor (ER) and erb-B2 receptor tyrosine kinase (ERBB)-associated signaling pathways, ferroptosis process and therapy response.

### Regulations of stemness and epithelial-mesenchymal transition (EMT)

**Fig. 2:**
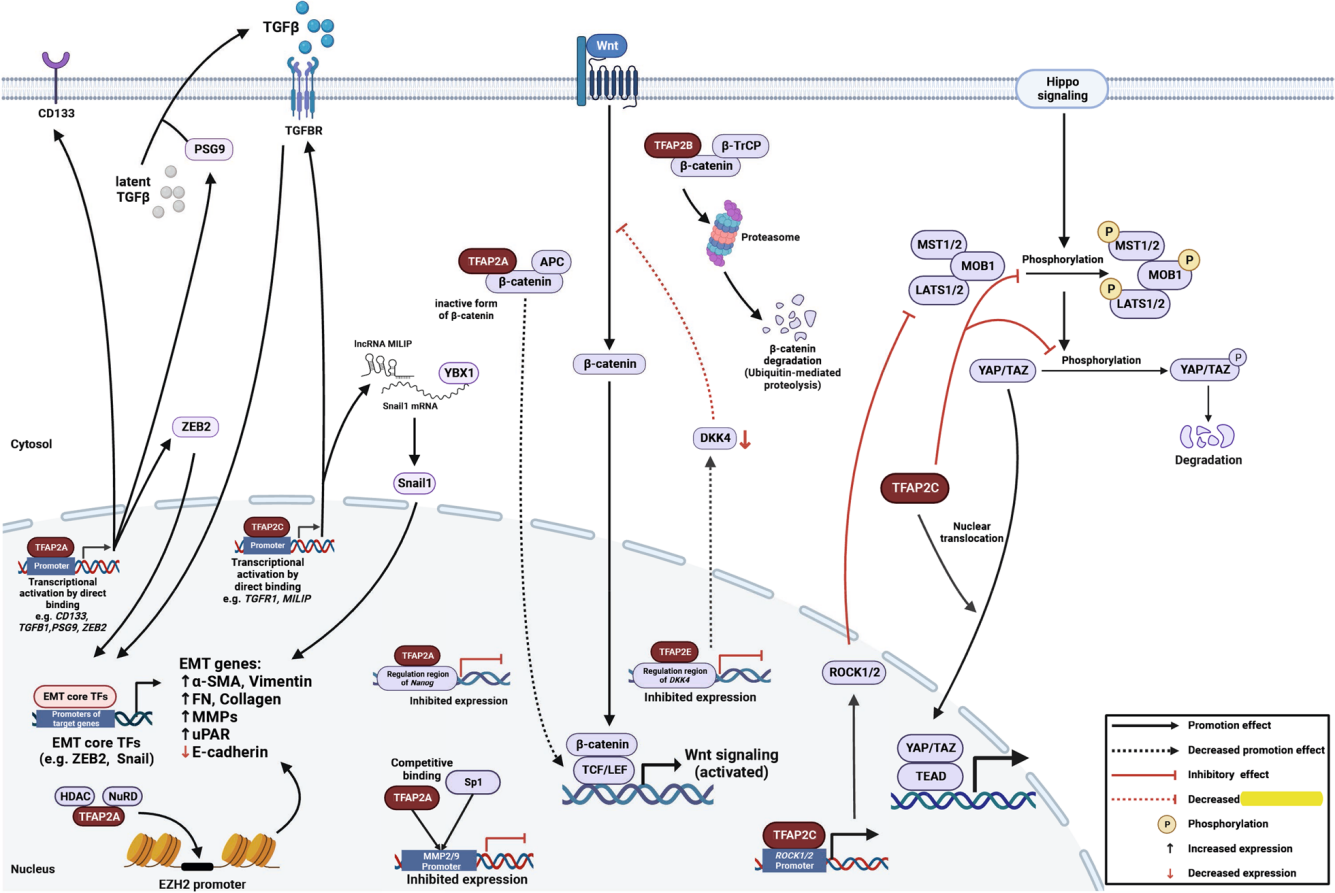
Graphic schematic of the regulations on stemness and EMT The role of TFAP2 in regulating stemness in cancer mainly through stemness-related genes, Wnt/β-catenin, TGF-β and EMT signaling pathways. Only validated direct protein–protein interaction and DNA/RNA–protein interaction are shown. (Created with BioRender.com)

Crucial regulation of TFAP2 members on stem cells during embryonic development is identified. Do they regulate “stem-like” phenotypes and gene signatures to promote stemness in cancer as well? Here, we summarize the role of TFAP2 in regulating stemness in cancer mainly through stemness-related genes, such as Wnt/β-catenin, transforming growth factor β (TGF-β) and EMT signaling pathways (Figure 2).

#### Stemness promotion

Among the TFAP2 family members, TFAP2A and TFAP2C are involved in regulating tumor stemness. Elevated expression is correlated with cancer stem-like phenotypes, promoted malignancy and poor prognosis. Mechanistically, TFAP2A directly binds to the CD133 promoter to ignite tumor stem-like properties in HCC cells and synergizes with periostin/TGFβ1 positive feedback loop to further boost tumor stemness [28]. In colorectal cancer, TFAP2C regulates stemness through various pathways. TFAP2C is restricted to the gonad, increased in testicular carcinoma, but downregulated in differentiated germ cells [29–31]. In colorectal carcinoma, TFAP2C enhances the expression of stemness-related factors, such as Nanog homeobox (NANOG), BMI1 proto-oncogene (BMI-1), POU class 5 homeobox 1 (OCT4) and SRY-Box transcription factor (SOX2), and the phenotypes of cancer stem cells. Furthermore, TFAP2C promotes yes associated protein 1 (YAP1) and tafazzin, phospholipid-lysophospholipid transacylase (TAZ) expression, known as transcriptional coactivators of Hippo signaling pathway; nuclear translocation of YAP/TAZ enhances TEA domain transcription factor (TEAD), but inhibits phosphorylation levels of macrophage stimulating 1/2 (MST1/2), large tumor suppressor kinase 1/2 (LATS1) and YAP. This indicates that the Hippo signaling pathway has been suppressed [32]. Additionally, TFAP2C binds to rho-associated coiled-coil containing protein kinase 1/2 (ROCK1/2) promoters to increase their expression, and ROCK1 and ROCK2 are suppressors of the Hippo signaling pathway by perturbing LATS activity [33].

Moreover, TFAP2B can interact with EP300 (histone acetyltransferase) to execute its critical transcriptional regulatory function in high-risk pediatric neuroblastoma [34].

#### EMT

Several studies have found TFAP2A and TFAP2C to be involved in the EMT process. Upregulated TFAP2A and TFAP2C tend to be observed more frequently in “stem-like” cancers. Yoana et al. discovered that TFAP2A and TFAP2C are crucial in the core transcription factors network that regulates EMT, and are likely involved in the early stages of EMT in breast cancer [35].

In TGFβ1-induced EMT, both TFAP2A and TFAP2C can directly activate TGF-β signaling-related genes to trigger EMT. TFAP2A directly binds to the promoter of *TGFB1* to induce the transcription of TGFB1 [36]. TFAP2C transcriptionally activates the *TGFBR1* expression. TGFBR1 subsequently activates downstream targets via a noncanonical pathway, including the P21-activated kinase 1 (PAK1), mitogen-activated protein kinase (MAPK) and PI3K/AKT pathways in NSCLC [37]. In addition, TFAP2A can transcriptionally activate pregnancy-specific glycoprotein 9 (*PSG9*) (binding latent TGF-β to release TGF-β) [38] to facilitate TGF-β-induced EMT. Besides, other EMT coactivators are regulated through transcriptional activation by directly binding to the promoter. TFAP2A is capable of binding promoters of zinc finger E-box binding homeobox 2 (*ZEB2*), a core regulator in EMT [35]. In clear cell renal cell carcinoma, TFAP2C binds to the MYC inducible lncRNA inactivating P53 (*MILIP*) promoter to increase MILIP expression (which enhances malignancy and metastasis through EMT). MILIP physically interacts with Y-box binding protein 1 (YBX1) and enhances the translational activation of another EMT core transcription factor, Snail family transcriptional repressor 1 (Snai1) and other factors, such as matrix metallopeptidase 2 (MMP2), N-cadherin and Vimentin, and decreases E-cadherin participation in EMT [39].

In addition, TFAP2A and TFAP2C can promote the expression of other oncogenes that synergize with metastasis and EMT processes. Studies have demonstrated that TFAP2A increases EZH2 expression by perturbing the activity of the NuRD complex to enhance hyperacetylation of the *EZH2* promoter region [13] and transcriptionally activate inositol 1,4,5-trisphosphate 3-kinase (ITPKA) and keratin 16 in lung adenocarcinoma (LUAD) [40, 41].

TFAP2C collaborating with LINC00857, boosts FAT atypical cadherin 1 (FAT1) expression (highly expressed in gastric cancer)to drive tumorigenesis and EMT [42].

Posttranslational modifications also influence the tumor-promoting and tumor-suppressing functions of TFAP2A and TFAP2C during the EMT process. SUMOylating of proteins is a type of posttranslational regulation that occurs by covalent and reversible binding of a small ubiquitin-like modifier (SUMO) to a target protein. In breast cancer, alteration of SUMOylation on TFAP2A and TFAP2C demonstrates potential transformation between luminal and basal breast cancer phenotypes and concordant gene signatures. TFAP2C maintains luminal phenotypes by regulating luminal gene signatures. Loss of TFAP2C and SUMOylation of TFAP2A induce CD44 expression and basal-related genes, which further boosts EMT and shifts to basal phenotypes (inhibiting luminal genes but promoting basal-associated genes) [43]. In colorectal and anaplastic thyroid cancers, similar SUMOylation on stemness regulation is also confirmed. SUMO inhibitors with SUMO-unconjugated TFAP2A reduce the cancer stem cell (CSC) population by suppressing CD44 and matrix metallopeptidase 14 (MMP14) expression to limit the stemness [43–45].

#### Suppression of stemness

TFAP2A and TFAP2B also exhibit repressive effects on cancer stemness.

An early study discovered that the presence of TFAP2A was associated with normal luminal differentiation of the prostate and that TFAP2A loss may be involved in the early occurrence of prostate adenocarcinoma [46]. In colorectal cancer cells, TFAP2A inhibits the Wnt/β-catenin signaling pathway. TFAP2A physically associates with APC, then the TFAP2A/APC/β-catenin complex shifts the nuclear β-catenin towards an inactive form and further reduces binding to T-cell factor/lymphoid enhancer factor (TCF/LEF) transcription factors, collectively disturbing Wnt/β-catenin signaling [47]. In glioma, TFAP2A disturbs the expression of stemness-related genes, such as *NANOG, SOX2* and *CD133*, via both directly binding the regulation region of *NANOG* and indirectly interfering with the IL6/JAK2/STAT3 signaling pathway [48]. TFAP2A overexpression in ovarian and colorectal cancer cells displays epithelioid morphology, possibly because TFAP2A binds to the promoters of E-cadherin and MMP2/9, resulting in increased E-cadherin expression but decreased MMP2/9 levels due to SP1 binding [49, 50].

TFAP2B is essential in normal renal development and the sympathetic nervous system. Downregulation of TFAP2B indicates potential dedifferentiation, EMT, and adipogenic transdifferentiation processes, which eventually result in the occurrence of clear cell renal cell carcinoma (ccRCC). From single-cell transcriptomic analyses, in neuroblasts and low-risk neuroblastoma, TFAP2B shows high transcription factor activity. Loss of TFAP2B is observed in high-risk neuroblastomas and progressive tumors [51–53]. In HCC, slow-growing luminal breast cancer, and cervical cancer, TFAP2B inhibits EMT and Wnt/β-catenin signaling to limit tumor growth and migration, which may be related to the Slug and Snail signaling pathways and loss of E-cadherin and low Ki67 [54–56]. Besides, by interacting with β-transducing repeat containing E3 ubiquitin protein ligase (β-TrCP), TFAP2B induces the proteasomal degradation of endogenous β-catenin [56].

TFAP2A and TFAP2B can directly interact with the retinoblastoma (Rb) protein, and TFAP2B interacts with the DEAH Box Protein 33 (DHX33), thereby mediating the expressions of B-cell lymphoma 2 (BCL2), MYC proto-oncogene protein (c-Myc) and E-cadherin by transcriptional activation of their promoters, which in turn arrests the cell cycle, induces apoptosis, and inhibits tumor growth, invasion and EMT in retinoblastoma and breast cancer [57–62].

TFAP2E, on the other hand, perturbs the Wnt/β-catenin signaling by transcriptionally inhibiting the expression of dickkopf Wnt signaling pathway inhibitor 4 (DKK4), an antagonist of Wnt signaling that occupies the Wnt coreceptor, in colorectal cancer [10, 63, 64].

Collectively, distinct effects of TFAP2 on stemness and EMT are observed even in the same type of cancer, implying that the underlying regulatory network is complex; therefore, further research is required to explain these controversies.

### Interaction between TFAP2 and the tumor microenvironment

**Fig. 3:**
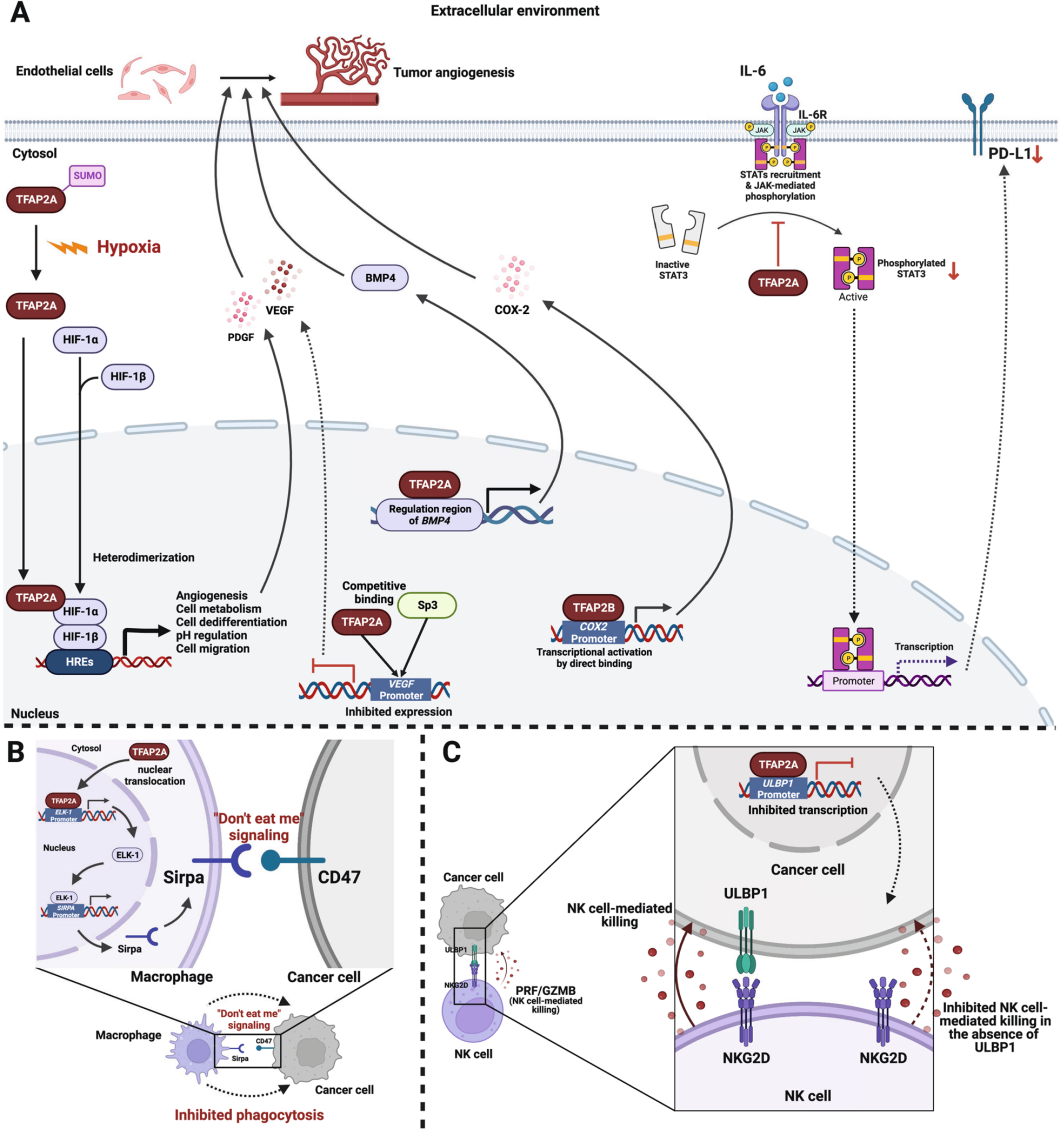
Interaction between TFAP2 and tumor microenvironment TFAP2 interact with tumor microenvironment through regulating angiogenesis and remodeling immune microenvironment. **A** Effects of TFAP2 on angiogenesis and immune related signaling pathways; **B** The regulation of TFAP2A on the interaction between cancer cells and macrophages; **C** The regulation of TFAP2A on the interaction between cancer cells and NK cells. Only validated direct protein–protein interaction and DNA/ RNA–protein interaction are shown. (Created with BioRender.com)

Based on published studies, TFAP2 interacts with the tumor microenvironment by regulating angiogenesis and remodeling the immune microenvironment (Figure 3).

#### Angiogenesis

Angiogenesis in cancer is a significant characteristic of malignancy that bridges the tumor cell and microenvironment.

In NSCLC, both TFAP2A and TFAP2B are implicated in promoting tumor-induced angiogenesis via platelet-derived growth factor receptor (PDGFR), vascular endothelial growth factor receptor (VEGFR) and TGF-β signaling pathways [65, 66]. Under hypoxic conditions, alteration of hypoxia inducible factor-1α (HIF-1α) in tumor cells and communication with microenvironment mediate angiogenesis, enhance malignancy, and promote chemoresistance. Dingbo et al. found that TFAP2A ignited HIF-1α-mediated VEGF/PEDF signaling activation in nasopharyngeal carcinoma [67]. Under hypoxic conditions, the decrease in TFAP2A SUMOylation triggers the transcriptional activity of HIF-1α to promote malignancy and angiogenesis [68]. Furthermore, exosomes from the hypoxic microenvironment, as well as miR-1246 and miR-10b-5p, target TFAP2A in normoxic glioma cells to promote angiogenesis and oncogenesis [69]. Overexpressed TFAP2B displays high microvessel density, angiogenesis and MMP2 expression in melanoma [70].

In addition to direct regulation of the HIF-1α/VEFG/PDGF pathway, TFAP2A transcriptionally activates bone morphogenetic protein 4 (*BMP4*) and heparan sulfate proteoglycan 2 (*HSPG2*), which activates endothelial cells by increasing VEGF-A/VEGFR2 and angiopoietin-1/Tie2 to further enhance angiogenesis [65]. TFAP2A promotes angiogenesis and glycolysis in bladder uroepithelial carcinoma (BLCA) by disrupting cartilage acidic protein 1 (CRTAC1) expression when exposed to hypoxia and malnutrition [71].

Notably, TFAP2A also exerts anti-angiogenesis effects in different tissues (inhibitory effects in prostate cancer). Competing with the transcriptional activator SP3, TFAP2A transcriptionally perturbs the *VEGF* promoter to limit VEGF expression [72].

#### Remodeling of the immune microenvironment

In terms of immune microenvironment remodeling, several bioinformatic studies have investigated the potential role of TFAP2A in regulating the extracellular matrix (ECM) and tumor immune microenvironment. TFAP2A acts as a potential transcription factor for some members of the ECM receptors and integrin subunit beta (ITGB) superfamily, potentially activating the signaling pathways of oncogenesis, ECM remodeling, EMT process, and immunosuppressive immune cell accumulation in pancreatic and colorectal cancer [73, 74]. TFAP2B binds to the cyclooxygenase 2 (*COX2*) promoter to trigger COX2 signaling, shaping a proinflammatory condition to increase malignancy in thyroid cancer [75].

Intriguingly, in colorectal cancer, TFAP2A could help to establish an inhibitory immune microenvironment by suppressing tumor-associated macrophage (TAM) phagocytosis. TFAP2A translocates from the cytoplasm into the nucleus to bind to the promoter region of the ETS Like-1 protein* (ELK-1)* gene and then activates its expression [76]. Afterward, inhibitory signaling is dramatically triggered by inducing the expression of signal regulatory protein α (SIRPα), which is known as a myeloid-specific immune checkpoint that engages in the "don't eat me" signaling pathway [77, 78]. UL16 binding protein 1 (ULBP1) is the ligand of NKG2D (also known as KLRK1, killer cell lectin like receptor K1), which facilitates natural killer (NK)-cell mediated cytotoxicity [79–81]. TFAP2A physically interacts with the *ULBP1* promoter to repress its expression, leading to escape from immunosurveillance. Besides, reduced binding activity between TFAP2A and the *ULBP1* promoter could also be induced by EGFR treatment [79]. Activated TFAP2A expression in glioma could negatively regulate the expression of O6-methylguanine methyltransferase (MGMT) and programmed death-ligand 1 (PD-L1) by inhibiting interleukin-6 (IL6)/Janus kinase 2 (JAK2)/STAT3 signaling pathways and also suppress the polarization of glioma-infiltrating microglia to M2 type macrophages, which show immunosuppressive characteristics [48]. In NSCLC, LncRNA PP7080 promotes the phosphorylation of TFAP2C, which then upregulates the expression of programmed cell death protein 6 (PDCD6) to promote cancer cell malignancy and to induce immune-suppressive tumor microenvironment by recruiting myeloid-derived suppressor cells [82].

### Regulations on the cell cycle and DNA damage repair

#### Cell cycle-related genes

TFAP2 suppresses tumor proliferation mediated through P21 (also known as CDKN1A, cyclin dependent kinase inhibitor 1A) regulation in several types of tumors, such as melanoma, breast cancer, colorectal cancer, pancreatic cancer and neuroblastoma [12, 83–89]. TFAP2E tends to act as a tumor suppressor. In neuroblastoma, adriamycin (ADR)-mediated induction of the CDK inhibitor P21 is upregulated in the absence of TFAP2E, indicating the implication of TFAP2E and P21 signaling pathway [89]. Further investigations revealed that in the pancreatic adenocarcinoma, TFAP2A inhibits cell cycle regulators, such as decreased cyclin dependent kinase 4 (CDK-4), cyclin dependent kinase 6 (CDK-6), cyclin-G1 and CDKN1C/P57, while increasing P27 (also known as CDKN1B, Cyclin Dependent Kinase Inhibitor 1B) by binding to the *P27* promoter to activate its expression by which cell cycle arrests [12, 87]. Additionally, both TFAP2A and TFAP2C can also bind to the *P21* promoter in breast and colorectal cancer cells to enhance P21 expression. Overexpression of TFAP2A and TFAP2C results in upregulated P21 but decreased cyclin D1 expression and subsequent decreased phosphorylation of Rb, leading to inactivation of adenoviral early region 2 binding factor (E2F), which stops the cell cycle [88].

However, studies have also shown the promoting effect of TFAP2 on the cell cycle. TFAP2C promoted lung cancer tumorigenesis by activating the cell cycle by upregulating miR-183 and miR-33a, which target *a-kinase anchoring protein 12 (AKAP12)* mRNA and *CDK6* mRNA [90]. Anna et al. found that TFAP2A could alter cell cycle progression by activating the PI3K/AKT cascade by targeting tissue transglutaminase (TGM), and participate in forming a complex with PI3K [91]. Interaction among TFAP2C, c-Myc and lysine demethylase 5B (KDM5B) can form a protein complex that promotes cell cycle progression by transcriptionally inhibiting *P21* expression, consequently resulting in tumor growth [92, 93]. In addition, in esophageal squamous cell carcinoma (ESCC), TFAP2C promotes the cell cycle, where TFAP2C activates the expression of polo-like kinase 1 (PLK1) by interacting with hematological and neurological expressed 1 like (HN1L) [94].

The controversial findings suggest that different protein/regulator interactions result in different regulatory outcomes.

#### TP53 (Tumor Suppressor Protein 53)

TFAP2A induces cell cycle arrest and apoptosis in colorectal cancer cells and breast cancer cells in a TP53-dependent and TP53-independent manner [85, 86]. Conversely, TP53 can help to loosen the chromatin structure of the regulatory region of the TFAP2A and TFAP2C promoters to increase chromatin accessibility that facilitates TP53 binding to their promoters to induce their expression and thus ultimately inhibit tumor proliferation. In addition, TFAP2A indirectly inhibits TP53 expression by upregulating the expression of MDM2, an upstream suppressor of TP53 [17].

#### DNA damage repair

TFAP2C inhibits GADD45B (growth arrest and DNA damage inducible β) and PMAIP1 (PMA-induced protein 1, pro-apoptotic subfamily within the BCL-2 protein family) expression in NSCLC cells to promote cell proliferation and cell motility [95].

In promoting oncogenesis, TFAP2B activates the human telomerase reverse transcriptase (hTERT) expression by specifically binding to its promoter in lung cancer cells rather than in normal cells. Activated telomerase prevents telomere shortening, leading to cell growth and reduced apoptosis [96, 97].

TFAP2A and TFAP2B bind the gene promoter of *USP22* (ubiquitin Specific Peptidase 22) to induce its expression and presumably promote the progression of NSCLC. USP22 can stabilize proteins, inhibit many TP53 host-protective functions and affect the DNA damage response [98, 99].

**Fig. 4:**
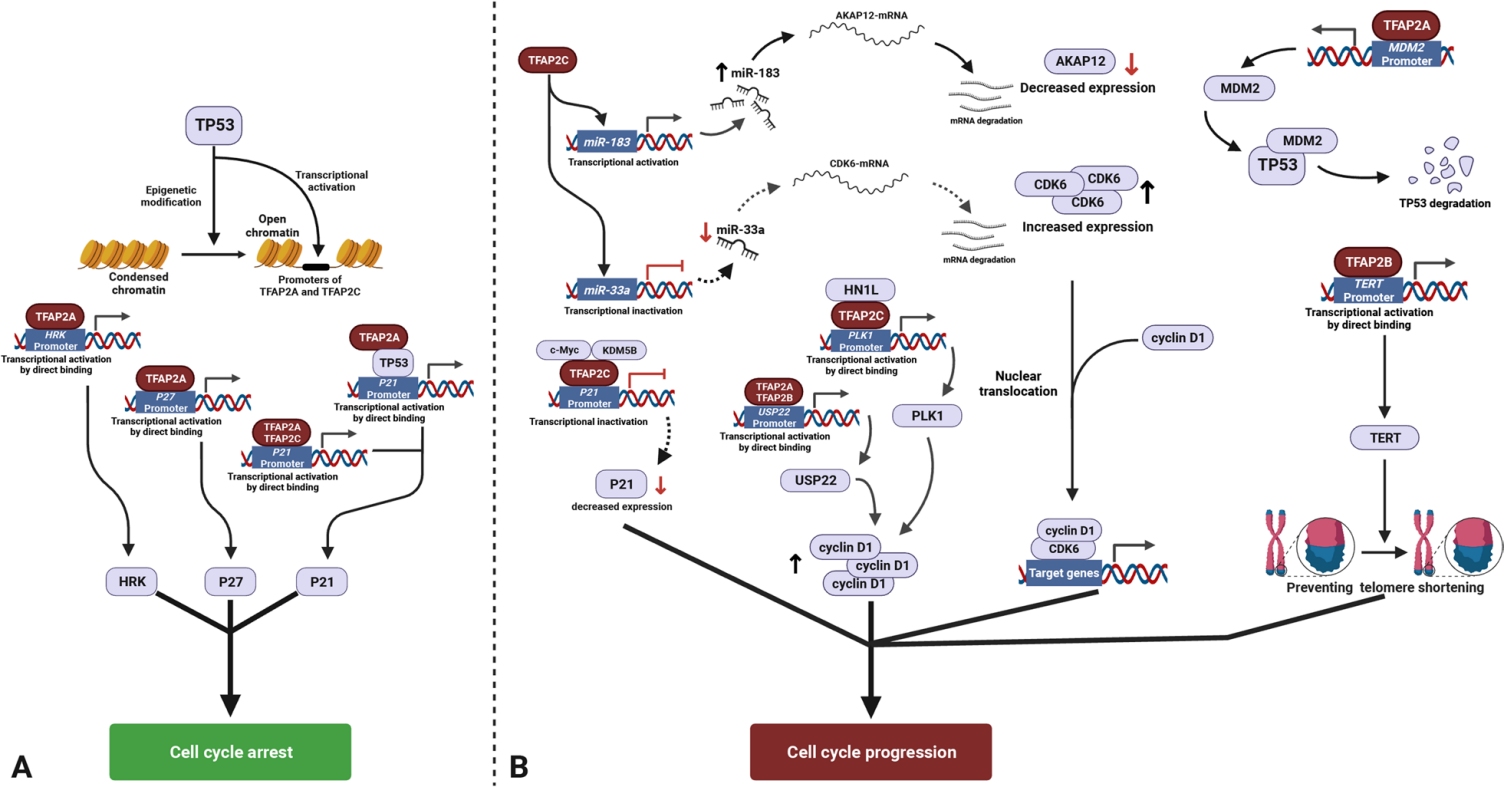
Summary of TFAP2 regulations on cell cycle and DNA damage repair **A** TFAP2 in regulating cell cycle arrest. **B** TFAP2 in regulating cell cycle progression. Only validated direct protein–protein interaction and DNA/RNA–protein interaction are shown. (Created with BioRender.com)

TFAP2A binds the harakiri (*HRK*) promoter to induce its expression. Elevated HRK leads to apoptosis and promotes the growth, invasion and metastasis of melanoma cells. (but in melanoma, this physical interaction is regulated by hypermethylation of the HRK promoter preventing binding of TFAP2A and synergically with the loss of TFAP2A) [100]. A graphic summary of the regulation of the cell cycle and DNA damage repair is shown in Figure 4.

### ER and ERBB-related signaling pathways

**Fig. 5:**
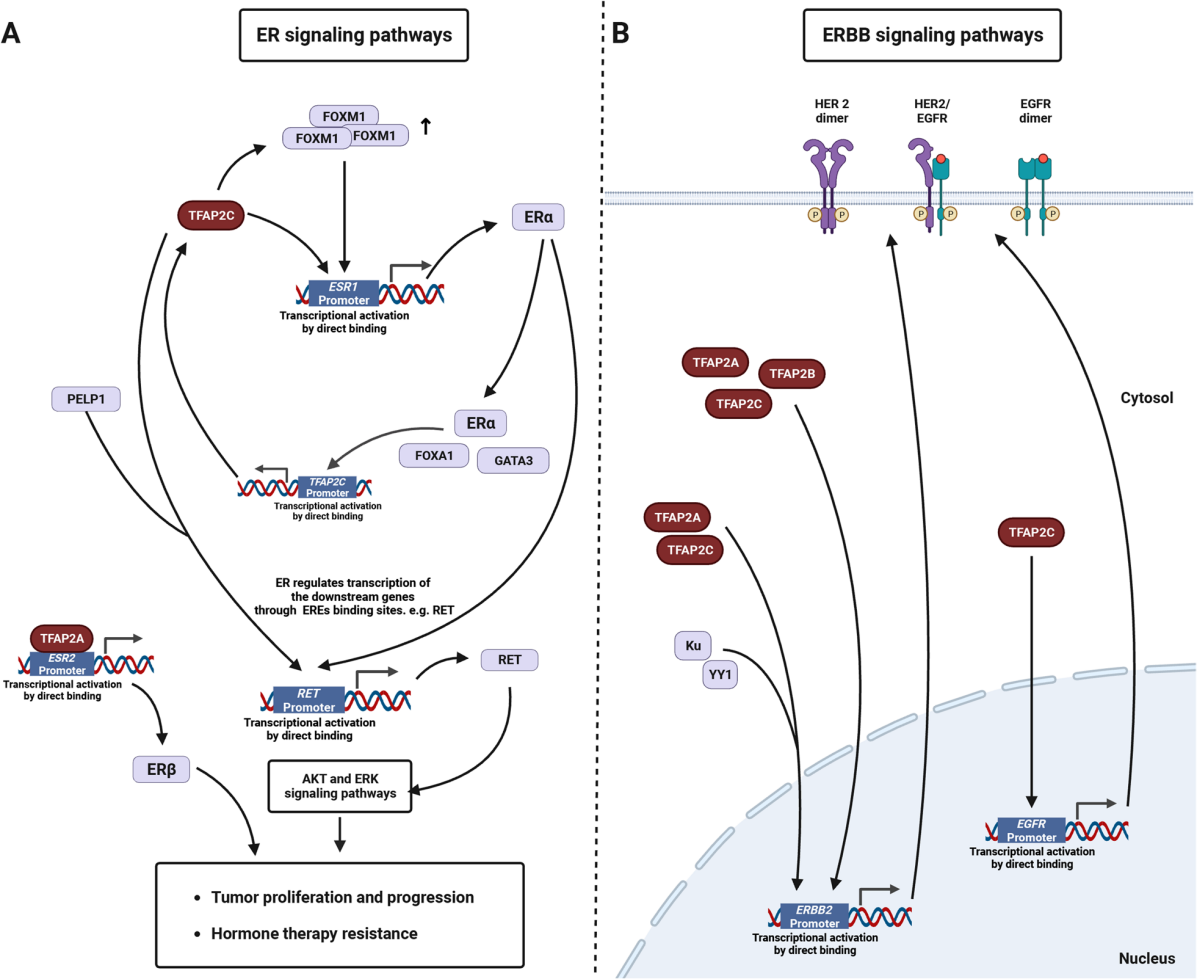
Regulation of TFAP2 on ER and ERBB signaling pathways **A** TFAP2 in regulating ER signaling pathway. **B** TFAP2 in regulating ERBB signaling pathway. Only validated direct protein–protein interaction and DNA/RNA–protein interaction are shown. (Created with BioRender.com)

Aberrant activation of ER and ERBB signaling pathways, which alters tumor malignancy and therapeutic response, is observed in various cancers, especially in breast cancer. Early investigations of breast cancer suggest that TFAP2 acts as a tumor-suppressive gene and that the absence of TFAP2 expression in the nucleus is correlated with poor prognosis and increased malignancy [101]. In addition, recent studies have specified that TFAP2 members regulate the ER- and ERBB-related pathways and the correlation with the prognosis of breast cancer patients. Here, we summarize the current studies about the role of TAFP2 members in the regulation of ER- and ERBB-related pathways. A graphic summary is shown in Figure 5.

#### ER-related signaling pathway

Several studies have shown that high TFAP2C expression is correlated with poor prognosis and resistance to anti-hormone therapy [102–104]. TFAP2C is indispensable for normal luminal cell development and retaining of luminal phenotypes in breast cancer. The loss of TFAP2C triggers EMT with phenotypic changes, which is related to the repressed expression of luminal-associated genes but induced expression of basal-associated genes [84, 105, 106]. Furthermore, it has been found that TFAP2C is implicated in the ERα and ERBB2 regulatory networks.

In detail, TFAP2C upregulates ER-related genes (*ESR1, FOXA1* and *GATA3*) to maintain the phenotypes of luminal breast cancer. TFAP2C enhances the expression of ERα by transcriptionally activating its promoter as well as indirectly by inducing forkhead box M1 (FOXM1) expression, which subsequently mediates ERα targeting genes. The loss of TFAP2C limits the mitogenic response to estrogen and estrogen-induced tumor proliferation [84]. ERα expression activated by TFAP2C is also regulated by CpG island methylation and histone 3 lysine 9 deacetylation. Demethylation and acetylation of the *ESR1* promoter by using methylation inhibitors and histone deacetylase inhibitors modify chromatin conformation and accessibility, thereby allowing TFAP2C to bind to it [107, 108].

Conversely, ER-related proteins (ERα, FOXA1 and GATA3) can bind regions of the transcription start site and transcriptional enhancer within the *TFAP2C* gene with an active chromatin conformation marked by high levels of H3K27Ac [104].

ERα transcriptionally activates Ret proto-oncogene (*RET*) via estrogen response elements (EREs) binding sites. In addition, TFAP2C can transcriptionally stimulate *RET* expression independently of ER expression in breast carcinoma [109, 110]. The TFAP2C-PELP1 (proline, glutamate and leucine rich protein 1) axis triggers the RET signaling (increased histone methylation by PELP1 facilitating TFAP2C binding to the *RET* promoter), which further induces downstream signaling such as the AKT and ERK pathways, in ER-positive breast cancer [111].

In prostate cancer, TFAP2A can bind to the *ESR2* promoter region to activate expression[112]. ERβ may exert its tumor promotion effects by enhancing cell invasion and metastasis [113–115].

#### ERBB-related signaling pathway

An expression trend between TFAP2 and ERBB2 in breast cancer was observed. TFAP2A, 2B and 2C are capable of directly binding to the *ERBB2* promoter to enhance its expression [116–119].

Studies on TFAP2A show its dual influences on breast oncogenesis, which possibly results from the differential transcriptional activity of TFAP2A isoforms on ERBB2 during the oncogenesis. Chiara et al. discovered that TFAP2A isoforms 1b and 1c are stronger transactivators of the *ERBB2* promoter than isoform 1a, while isoform 1a acts as a repressor [120].

TFAP2C increases ERBB-related genes (*EGFR, ERBB2, ERBB3*), which mediate oncogenesis in ERBB2/HER2-amplified breast cancer [106, 108, 117–119, 121–123]. Mechanically, TFAP2A and TFAP2C are capable of binding the *ERBB2* promoter, and in collaboration with other transcription factors, such as Ku protein and Yin Yang 1, they significantly promote ERBB2 expression in breast cancer cells [116, 117, 124]. Conversely, krüppel‐like factor 14 (KLF14) elevates the miR-1283 level, which inhibits the TFAP2C expression by targeting the 3'-UTR of *TFAP2C* mRNA [125].

Additionally, TFAP2C directly interacts with the *EGFR* gene to promote its expression, resulting in luminal breast cancer cell proliferation and an improved therapeutic effect of TKIs (Vandetanib) [106, 126].

### Ferroptosis process

**Fig. 6:**
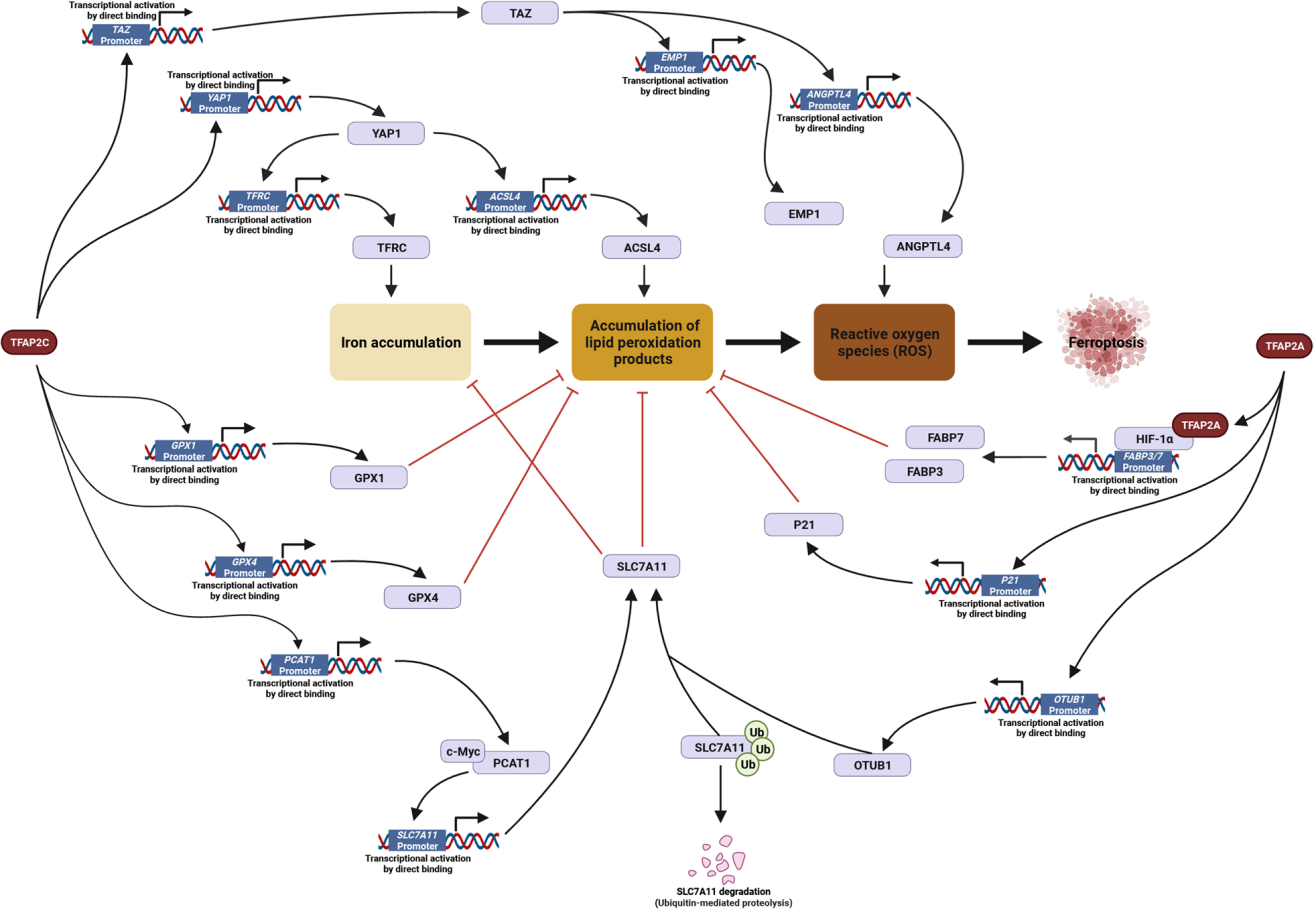
The Regulations of TFAP2 on ferroptosis process Ferroptosis is characterized by abnormal iron metabolism and accumulation, lipid biosynthesis and peroxidation, and ROS (reactive oxygen species) in cell, and eventually regulates the process of cell death. TFAP2 members multidimensionally involve ferroptosis process by directly regulating ferroptosis-related proteins.Only validated direct protein–protein interaction and DNA/RNA–protein interaction are shown. (Created with BioRender.com)

Ferroptosis, a dedicated-regulated process of cell death, is characterized by abnormal iron metabolism and accumulation, lipid biosynthesis and peroxidation, and ROS (reactive oxygen species) in cells. TFAP2C is reported to be mainly involved in preventing ferroptosis [127, 128] by directly promoting epidermal growth factor receptor (*EGFR*) [126], glutathione peroxidase 1 (*GPX1*) [129], glutathione peroxidase 1 (*GPX4*) [130], *YAP1* [32] and *TAZ* [32], while repressing *P21* [92]. These factors are involved in several steps in ferroptosis regulation (Figure 6).

In breast cancer, TFAP2C regulates the sensitivity to peroxide by mediating the expression of *GPX1*. GPX1 expression is enhanced by TFAP2C via the TFAP2 regulatory region within the *GPX1* promoter (methylation of the CpG island within the TFAP2 regulatory region leads to reduced binding activity and decreased GPX1 expression) [129]. Elevated GPX1 expression levels are linked to increased tumorigenesis and progression by regulating ROS [131, 132].

Indirectly, it is observed in progressive pancreatic cancer, and TFAP2A can bind the promotor region of OTU Deubiquitinase (*OTUB1*) to upregulate its expression. OTUB1 is a type of deubiquitinating enzyme that interacts with solute carrier family 7 member 11 (SLC7A11) to prevent ubiquitinated degradation of SLC7A11 [133]. In bladder cancer, TFAP2C transcriptionally increases prostate cancer associated transcript 1 (*PCAT1*) expression, which physically interacts with c-Myc to transcriptionally activate SLC7A11 expression. During ferroptosis, SLC7A11 inhibits iron accumulation and subsequent oxidative damage [134].

Besides, TFAP2C and SP1 are implicated in the protection of cerebral hemorrhagic injury by inducing GPX4 expression to prevent lipid peroxidation, a crucial step in ferroptosis, in tissue injury [130].

### Unclassified pathways regulated by TFAP2

Regarding TFAP2A, in several cancers, elevated TFAP2A expression is linked to enhanced proliferation, migration and invasion abilities. In neuroblastoma, the expression of TFAP2A is related to poor differentiation and advanced tumor stage. Reduced proliferation and induced differentiation were observed when TFAP2A was knocked down [135].

TFAP2A binds to the promoters of tectonic family member 1 (*TCTN1*) and amyloid precursor protein (*APP*)to induce malignancy (proliferative, migratory and invasive capacity) in the oral squamous cell carcinoma (OSCC) [136, 137].

In lung squamous cell carcinoma (LUSC) and pancreatic adenocarcinoma (PAAD), TFAP2A-induced SLC2A1-AS1 (solute carrier family 2 member 1-antisense RNA 1) promotes tumor malignancy. TFAP2A triggers the transcription of *SLC2A1-AS1* via direct binding to the promoter of the *SLC2A1-AS1*-encoding gene [138].

In endometrial carcinoma, TFAP2A transcriptionally induces small nucleolar RNA host gene 16 (*SNHG16*) expression by binding to the promotor, leading to tumor growth and glycolysis [139].

For TFAP2C, in bladder cancer, TFAP2C expression is elevated, which is associated with enhanced malignancy and cisplatin resistance. In bladder cancer, in cooperation with PPARɣ inactivation and other transcription factors, TFAP2A and TFAP2C overexpression induced the shift from the luminal subtype to the basal-squamous transition during tumor progression [140].

### Therapeutic response/indicator

#### Classic broad cytotoxic anti-tumor drug

Based on current studies, TFAP2 regulates the therapeutic response to classic broad cytotoxic anti-tumor drugs, including gemcitabine, adriamycin, cisplatin, docetaxel and 5-fluorouracil (5-FU).

TFAP2A tends to improve sensitivity, while TFAP2C tends to induce the resistance to chemotherapy. For example, overexpressed TFAP2A could increase the sensitivity to gemcitabine in pancreatic cancer [87] and increase chemosensitivity to adriamycin and cisplatin in lung carcinoma [85]. Downregulating TFAP2A in bladder cancer cells induces cisplatin resistance [141]. The decreased expression of *TFAP2A* resulting from hypermethylation leads to chemoresistance to adriamycin and cisplatin in breast cancer [142]. In a study of TFAP2C, cisplatin treatment was found to enhance the activation of EGFR and nuclear factor kappa B (NF-κB) signaling, while the loss of TFAP2C repressed this process. TFAP2C is elevated in bladder cancer and testicular germ cell tumor to mediate cisplatin resistance and increase malignancy [143]. TFAP2C is also implicated in docetaxel resistance by promoting the cell cycle in ESCC and by inhibiting ferroptosis in bladder cancer [94, 134].

5-FU is another anti-tumor agent; TFAP2C mediates the chemotherapy response of colorectal cancer (CRC) cells to 5-FU [32]. In addition, several investigations on TFAP2E have shown a tumor suppressive influence, and decreased expression levels indicate promoted oncogenesis, stemness and chemoresistance in several types of cancers, including colorectal cancer, gastric cancer, neuroblastoma and skin neoplasia [10, 144–146]. Several studies have revealed that hypermethylation of the *TFAP2E* gene is correlated with early tumor stages, low invasion, reduced lymph node metastasis, and favorable prognosis in colorectal cancer [146–148]. Ebert et al. first found chemoresistance mediated by *TFAP2E* gene methylation and TFAP2E-DKK4 expression in colorectal cancer. *TFAP2E* hypermethylation is correlated with decreased *TFAP2E* expression, which promotes DKK4 expression and further induces fluorouracil chemoresistance (A direct binding to the TFAP2E protein to the *DKK4* promoter results in inhibited DKK4 expression) [10]. DKK4 has been demonstrated to be involved in fluorouracil resistance in colorectal cancer [63, 64].

Several studies have revealed that *TFAP2E* methylation status is potentially associated with the chemotherapy response to 5-FU treatment in colorectal carcinoma. Besides, in gastric cancer, the methylation status of *TFAP2E* is also reported to be involved in the 5-FU response, and epigenetic drugs that target DNA methylation are able to rescue TFAP2E-mediated 5-FU sensitivity in gastric cancer [144, 149, 150]. Oscar et al., however, argued that when unifying tumor stage and therapy regimen in their cohort, *TFAP2E* hypermethylation is a stochastic event and not significantly correlated with its expression. Additionally, neither TFAP2E expression nor *TFAP2E* methylation status is a predictor of the response to 5FU chemotherapy. However, it should be noted that *TFAP2E* methylation is an independent predictor of early recurrence, especially for stage II CRC patients [151].

No consensus has been reached that *TFAP2E* gene hypermethylation correlates with the loss of its protein expression at the tissue level across all stages of the tumor. The controversy indicates that the influences of the complex cellular regulatory network and undiscovered interactions with the tumor microenvironment on TFAP2E are indispensable.

#### Anti-hormone therapy

TFAP2C is associated with unfavorable clinical outcomes and decreased response to anti-hormone therapy, such as tamoxifen and fulvestrant, in breast cancer. As summarized before, in breast cancer, TFAP2C regulates ER expression and plays an essential role in mediating the hormone response by regulating multiple pathways of ER-related signaling. TFAP2C transcriptionally activates the expression of ERα. Additionally, TFAP2C regulates the expression of downstream genes of ERα, which are essential effectors in anti-hormone therapy. Therefore, an inhibited mitogenic response to estrogen treatment and decreased hormone-responsive tumor growth are observed when TFAP2C expression is downregulated [84, 102, 103, 152].

#### Targeted drugs

In recent years, tyrosine kinase inhibitors (TKIs) have been recommended as the first-line treatment for serval cancers. The classic targeted pathways include VEGFR, fibroblast growth factor receptor (FGFR), PDGFR, and c-kit (KIT proto-oncogene). In NSCLC, TFAP2A is implicated in promoting tumor-induced angiogenesis and inducing resistance to the anti-angiogenic effect of anlotinib via PDGFR, TGF-β, and VEGFR signaling pathways [65]. LINC00160 recruits TFAP2A to increase serum amyloid A1 (*SAA1*) expression by binding its promoter. Upregulated SAA1 leads to Sunitinib resistance and JAK/STAT signal pathway activation in renal cell carcinoma [153]. TFAP2C directly interacts with the *EGFR* gene to promote its expression, resulting in luminal breast cancer cell proliferation and an improved therapeutic effect of TKI (Vandetanib) [126].

TFAP2 can also be a predictor of chemotherapy response. For example, TFAP2A could be regarded as a biomarker for predicting the therapy response of PI3K inhibitors in colorectal cancer [91]. Increased TFAP2C and reduced CD44 expression could be predictive of neoadjuvant chemotherapy in breast cancer [121].

Targeting transcription factors has been an emerging concept for cancer treatment in recent years. A few studies demonstrated that targeting TFAP2 can be a promising therapy though, emerging data on TFAP2-focused targeted therapy suggests its potential in cancer therapy [154–156]. Further in-depth investigations are required to extend and validate this concept.

## Factors that alter TFAP2 expression

**Fig. 7:**
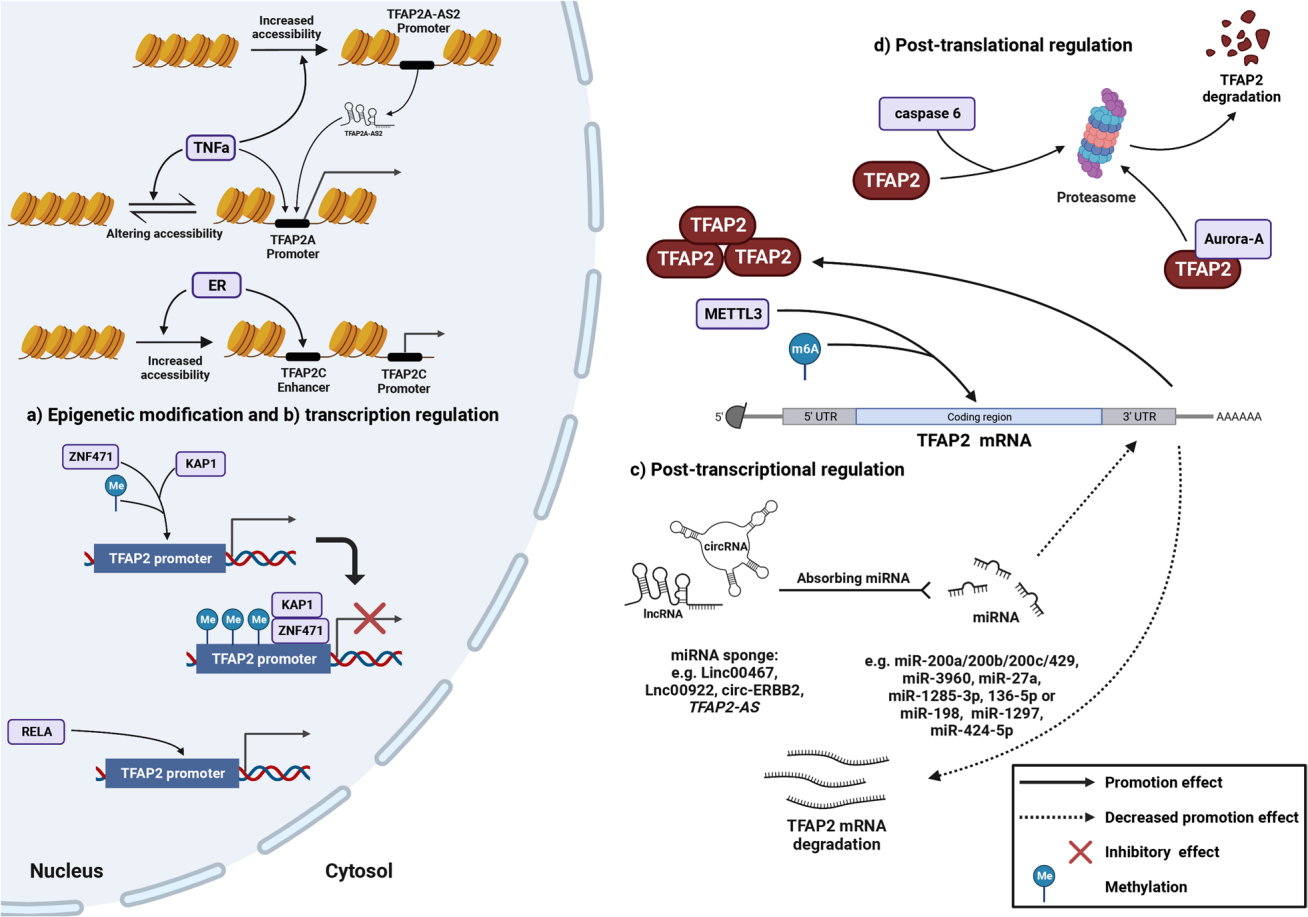
Regulatory modes on TFAP2 expression The TFAP2 expression is altered by **a** epigenetic modification to alter the accessibility of TFAP2 enhancer or promoter; **b** transcriptional regulation, e.g., directly interacting with cis-regulatory element of TFAP2 gene; **c** post-transcriptional regulation, e.g., modifying the stability of TFAP2 mRNA and **d** posttranslational regulation, e.g., inducing accumulation or degradation of TFAP2 protein. Only validated direct protein–protein interaction and DNA/RNA–protein interaction are shown. (Created with BioRender.com)

The TFAP2 protein expression could be regulated by epigenetic modification on the *TFAP2* gene, mediation with other regulators at the transcriptional level and post-transcriptional regulation (Figure 7).

### Epigenetic modification

Epigenetic alterations are observed in TFAP2 members, including histone acetylation and DNA methylation. As mentioned above, in breast cancer, ER-related genes inversely induce TFAP2C expression by transcriptional activation and reliving chromatin conformation marked by high levels of histone H3 lysine 27 acetylation (H3K27Ac) [104]. Using single-cell transposase accessible chromatin sequencing, Liu et al. have discovered that tumor necrosis factor (TNFα)-induced changes in accessibility vary depending on different tissues. For instance, in cholangiocarcinoma cells, TNFα induces increased accessibility of the *TFAP2A* promoter, while it is reduced in gallbladder cancer cells [157].

In addition, DNA methylation is an important regulatory mechanism that alters TFAP2 expression and is related to tumor malignancy and therapy response. Epigenetic modification is observed in multiply loci, including promoters, exons and introns within the *TFAP2* gene. In human melanoma, TFAP2A expression is regulated by its promoter CpG methylation. Increased promoter methylation reduces TFAP2A expression to promote metastasis [158]. During breast cancer evolution, several CpG sites within the *TFAP2A* gene are hypermethylated; in particular, an approximately 300bp area at the 3' end of exon 1 can be obviously correlated with transcriptional inactivation and further distinguish tumors from normal breast tissue [159]. In primary neuroblastomas, low TFAP2B expression is also significantly related to CpG methylation of the *TFAP2B* locus [51].

### Transcription level

At the transcription level, zinc-finger protein 471 (ZNF471) suppressed the expression of TFAP2A in gastric cancer. In addition, ZNF471 could even recruit KAP1 (also known as tripartite motif containing 28, TRIM28) to the promoter of the *TFAP2A* gene, which induces histone 3 lysine 9 trimethylation (H3K9me3) enrichment for further transcriptional repression and inhibition [160], consequently inhibiting cell proliferation, migration, invasion and xenograft tumorigenesis but inducing apoptosis. RELA was found directly interact with the cis-regulatory region of TFAP2A, to upregulate the TFAP2A expression in the oral squamous cell carcinoma (OSCC). After activation, multiple malignant phenotypes were promoted [161].

### Post-transcriptional and post-translational regulation

Post-transcriptionally, N6-methyladenosine (m6A) on *TFAP2C* mRNA is modified by methyltransferase-like protein 3 (METTL3), which increases the stability of *TFAP2C* mRNA, consequently upregulating TFAP2C expression [162]. Additionally, microRNA interacts with the target mRNA 3' untranslated region (UTR) to affect its stability and regulate its expression. This kind of regulation is also widely observed in *TFAP2* mRNA. For example, miR-200a targets *TFAP2C* mRNA [163], miR-200b suppresses *TFAP2A* expression in cholangiocarcinoma [36], miR-3960 suppresses *TFAP2A* mRNA in the pancreatic cancer [164], and miR-27a inhibits *TFAP2B* mRNA in HCC [55]. Additionally, single nucleotide polymorphism (SNP) site variation decreases the binding activity of miR-200b/200c/429 to the 3' UTR of *TFAP2A* mRNA, which upregulates TFAP2A expression and increases cisplatin sensitivity [165]. Long noncoding RNAs (lncRNAs) and circular RNAs (circRNAs) can both act as competing endogenous RNAs (ceRNAs) to absorb miRNAs, which further regulates target gene expression. For example, Linc00467 acts as a ceRNA to adsorb miR-1285-3p to mediate *TFAP2A* expression in head and neck squamous cell carcinoma (HNSCC) [166]. circ-ERBB2 acts as a ceRNA for miR-136-5p or miR-198 to relieve the repressive influence of miR-136-5p or miR-198 on *TFAP2C* in breast cancer [167]. Besides, antisense lncRNAs can promote the transcription of their counterpart sense mRNAs by acting as miRNA sponges. *TFAP2A-AS1* acts as a miR-1297 sponge, which inhibits the TFAP2A expression in OSCC [168].

Cooperation between TFAP2 and lncRNA was found to regulate TFAP2 expression and tumor malignancy. In osteosarcoma, there is a positive feedback loop between TFAP2C and Lnc00922. TFAP2C promotes the transcription of Lnc00922; in addition, Lnc00922 acts as a sponge of miR-424-5p, which is able to bind the *TFAP2C* mRNA 3' UTR to inhibit its translation. Consequently, the expression of TFAP2C is maintained in osteosarcoma to enhance malignancy and doxorubicin resistance [169].

The expression levels of TFAP2A and TFAP2C are reduced post-translationally during TNFα-induced cell apoptosis, during which TFAP2A is cleaved by caspase 6 and degraded by the proteasome in the breast cancer cells [170]. Aurora-A, a member of the Aurora kinase family, is found interacting with TFAP2A, resulting in the decreased stability of TFAP2A protein by ubiquitin-dependent proteasomal degradation [171].

## Conclusions

We summarize the current state of knowledge regarding the role of TFAP2 members in carcinogenesis. TFAP2 regulates downstream molecules primarily at the transcriptional level, but also through epigenetic modification and post-translational regulation. The role of TFAP2 in tumorigenesis involves the regulation of stemness and EMT, interactions between TFAP2 and the tumor microenvironment, the cell cycle and DNA damage repair, ER- and ERBB2-related signaling pathways, ferroptosis and the therapeutic response. However, there are still several critical pathways and key biological processes that play important roles in the development and progression of tumors, such as autophagy, pyroptosis, immunogenic cell death, and metabolic abnormalities et al. They have not been explicitly described when the TFAP2 expression is altered. For future research, it remains necessary to further elucidate their roles in cancers. Additionally, research on TFAP2D is currently limited, and further studies are needed to extend our understanding of its role in cancers.

Moreover, determining whether TFAP2 acts as a tumor promoter or suppressor is challenging because TFAP2's effects on tumorigenesis vary across tissues and downstream pathways. Besides, a multitude of regulatory mechanisms are involved in the TFAP2 network. Current studies have explained many phenotypes and dissected the underlying mechanisms, but more studies are still needed to further uncover the factors that trigger the alteration of TFAP2 expression and the regulatory networks connecting TFAP2 and other molecules.

Current studies were most on solid tumors; there is a lack of investigations into hematologic malignancies. Furthermore, most studies on TFAP2 focus on cancer cells themselves. However, it is still required to describe the interaction between cancer cells with altered TFAP2 expression and other stromal and immune cells.

Moreover, the role of TFAP2 in targeted therapy is often underestimated, despite the fact that some members, particularly TFAP2D, can be investigated for their druggability due to their specific protein structure. Recent data on TFAP2-based targeted therapy suggests its potential in cancer therapy. Further in-depth investigations are required to explore cancer therapy by targeting key transcription factors. In some targeted therapy and chemotherapy in cancers, TFAP2 can act as a predictor or regulator, but its clinical significance is often overlooked. More studies are needed to link TFAP2 to its clinical significance together, which can help eventually utilize TFAP2 in clinical and translational settings.

## Supplementary Information

**Additional file 1: Figure S1.** PRISMA flow diagram for literature identifying and screening.

## Data Availability

Not applicable.

## Abbreviations

ACSL4: Acyl-CoA Synthetase Long Chain Family Member 4
AKAP12: A-Kinase Anchoring Protein 12
AKT: AKT Serine/Threonine Kinase, also known as Protein Kinase B (PKB)
ANGPLT4: Angiopoietin Like 4
APC: Adenomatosis Polyposis coli Tumor Suppressor
APP: Amyloid Precursor Protein
BCL2: B-cell Lymphoma 2
BLCA: Bladder Uroepithelial Carcinoma
BMI-1: BMI1 Proto-oncogene
BMP4: Bone Morphogenetic Protein 4
β-TrCP: Beta-Transducing Repeat Containing E3 Ubiquitin Protein Ligase
ccRCC: Clear Cell Renal Cell Carcinoma
CDK6: Cyclin Dependent Kinase 6
c-Myc: MYC Proto-Oncogene Protein
COX2: Cyclooxygenase 2
CRC: Colorectal Cancer
CRTAC1: Cartilage Acidic Protein 1
CSC: Cancer Stem Cell
DKK4: Dickkopf WNT Signaling Pathway Inhibitor 4
DHX33: DEAH Box Protein 33
E2F: Early Region 2 Binding Factor
ECM: Extracellular Matrix
EGFR: Epidermal Growth Factor Receptor
ELK1: ETS Like-1 Protein
EMP1: Epithelial Membrane Protein 1
EMT: Epithelial-mesenchymal Transition
ER: Estrogen Receptor
EREs: Estrogen Response Elements
ERK: Extracellular-signal Regulated kinase
ERBB2: Erb-B2 Receptor Tyrosine Kinase 2 or Human Epidermal Growth Factor Receptor-2 (HER2)
ESCC: Esophageal Squamous Cell Carcinoma
EZH2: Enhancer of Zeste 2 Polycomb Repressive Complex 2 Subunit
FABP3/7: Fatty Acid Binding Protein 3/7
FAT1: FAT atypical cadherin 1
FOXA1: Forkhead Box A1
FOXM1: Forkhead Box M1
FUT8: Fucosyltransferase 8
GADD45B: Growth Arrest and DNA Damage Inducible β)
GATA3: GATA Binding Protein 3
GOLPH3: Golgi Phosphoprotein 3
GPX1/4: Glutathione Peroxidase 1/4
GZMB: Granzyme B
H3K9me3: Histone 3 Lysine 9 Trimethylation
H3K27Ac: Histone H3 Lysine 27 Acetylation
HDAC: Histone Deacetylase
HIF-1α/β: Hypoxia Inducible Factor 1 Subunit α/β
HN1L: Hematological and Neurological Expressed 1 Like
HNSCC: Head and Neck Squamous Cell Carcinoma
HREs: Hypoxia-Response Elements
HRK: Harakiri or BCL2 Interacting Protein
HSPG2: Heparan Sulfate Proteoglycan 2
IL-6(R): Interleukin-6 (Receptor)
ITPK: Inositol 1,4,5-trisphosphate 3-kinase
LATS1/2: Large Tumor Suppressor Kinase 1/2
LUAD: Lung Adenocarcinoma
LUSC: Lung Squamous Cell Carcinoma
JAK: Janus Kinase
K63-Ub: K63 Chain-linked Ubiquitination
KDM5B: Lysine Demethylase 5B
KLF14: Krüppel‐like Factor 14
m6A: N6-methyladenosine
METTL3: Methyltransferase-like Protein 3
MAPK: Mitogen-activated Protein Kinase
MGMT: O6-methylguanine Methyltransferase
MILIP: MYC Inducible LncRNA Inactivating P53
MMP2: Matrix Metallopeptidase 2
MMP14: Matrix Metallopeptidase 14
MOB1: MOB Kinase Activator
MST1/2: Macrophage Stimulating 1/2
MTA1: Metastasis-Associated Protein 1
MUC4: Mucin 4
NANOG: Nanog Homeobox
NF-κB: Nuclear Factor Kappa B
NK cell: Natural killer cell
NKG2D: also known as KLRK1, Killer Cell Lectin Like Receptor K1
NSCLC: Non-small Cell Lung Cancer
NuRD: Nucleosome Remodeling and Deacetylase
OCT4: POU Class 5 Homeobox 1
OSCC: Oral Squamous Cell Carcinoma
OTUB1: OTU Deubiquitinase
P21: also known as CDKN1A, Cyclin Dependent Kinase Inhibitor 1A
P27: also known as CDKN1B, Cyclin Dependent Kinase Inhibitor 1B
PAAD: Pancreatic Adenocarcinoma
PAK1: P21-activated Kinase 1
PAR1: Protease-activated Receptor-1
PCAT1: Prostate Cancer Associated Transcript 1
PDCD6: Programmed Cell Death Protein 6
PDGF: Platelet Derived Growth Factor
PDGFR: Platelet Derived Growth Factor Receptor
PD-L1: Programmed Cell Death 1 Ligand 1
PELP1: Proline, Glutamate And Leucine Rich Protein 1
PHLPP1: PH Domain and Leucine Rich Repeat Protein Phosphatase 1
PLK1: Polo-like Kinase 1
PMAIP1: PMA-induced Protein 1
PPARβ/δ: Peroxisome Proliferator-activated Receptors β/δ
PRF: Perforin
PSG9: Pregnancy-specific glycoprotein 9
Rb: Retinoblastoma
RET: Ret Proto-Oncogene
ROCK1/2: Rho Associated Coiled-Coil Containing Protein Kinase 1/2
SAA1: Serum Amyloid A1
SIRPA: Signal Regulatory Protein Alpha
SLC2A1-AS1: Solute Carrier Family 2 Member 1-antisense RNA 1
SLC7A11: Solute Carrier Family 7 Member 11
Snail: Snail Family Transcriptional Repressor 1
SNHG16: Small Nucleolar RNA Host Gene 16
SNP: Single Nucleotide Polymorphism
SOX2: SRY-box Transcription Factor 2
SOX8: SRY-box Transcription Factor 8
SP1: Specificity Protein 1
SP3: Specificity Protein 3
STAT3: Signal Transducer And Activator of Transcription 3
SUMO: Sumoylation
TAM: Tumor-associated Macrophages
TAZ: Tafazzin, Phospholipid-Lysophospholipid Transacylase
TCF/LEF: T-cell factor/lymphoid enhancer factor transcription factors
TCTN1: Tectonic Family Member 1
TEAD: TEA Domain Transcription Factor
TERT: Telomerase Reverse Transcriptase
TFRC: Transferrin Receptor
TFs: Transcription Factors
TFAP2: Transcription factors family activator protein 2
TGF-β: Transforming Growth Factor Beta
TKIs: Tyrosine Kinase Inhibitors
TNFα: Tumor Necrosis Factor α
TP53: Tumor Suppressor Protein 53
TRIM29: Tripartite Motif-containing 29
TRIM37: Tripartite Motif-containing 37
Ub: Ubiquitination
ULBP1: UL16 Binding Protein 1
USP22: Ubiquitin Specific Peptidase 22
UTR: Untranslated Region
VEGF: Vascular Endothelial Growth Factor
VEGFR: Vascular Endothelial Growth Factor Receptor
WWOX: WW Domain Containing Oxidoreductase
YAP: Yes Associated Protein
YBX1: Y-box Binding Protein 1
YY1: Yin Yang 1
ZEB2: Zinc Finger E-Box Binding Homeobox 2
ZNF471: Zinc-finger Protein 471
