# Supplementary material for: Crucial role of the transcription factors family activator protein 2 in cancer: current clue and views
Source: J Transl Med. 2023 Jun 8;21:371. doi: 10.1186/s12967-023-04189-1 (PMC10249218; doi:10.1186/s12967-023-04189-1)
Supplement: Supplementary file 1 — Additional file 1: Figure S1. PRISMA flow diagram for literature identifying and screening. [file 12967_2023_4189_MOESM1_ESM.pdf]

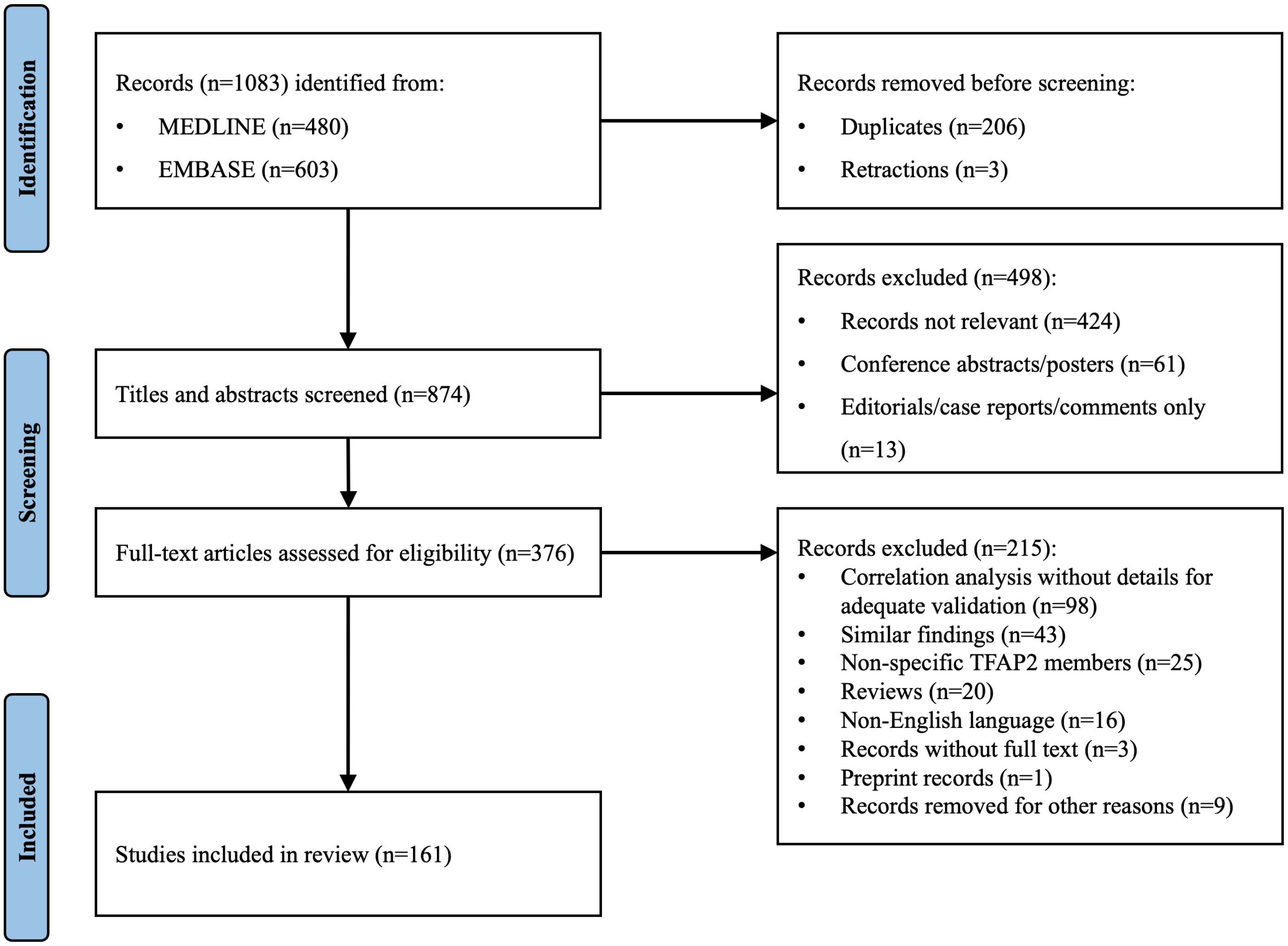

Figure S1: PRISMA flow diagram for literature identifying and screening

The PRISMA flow diagram illustrates the process of identifying and screening records pertaining to the involvement of TFAP2 in cancer. The initial search yielded 1083 records (480 MEDLINE; 603 EMBASE), which underwent deduplication and retraction to produce a total of 874 records. Two independent reviewers then screened these records by title and abstract. Of the resulting 376 eligible records, 424 irrelevant records, 61 conference abstracts/posters, and 13 editorials/case reports/comments lacking experimental data were excluded. The remaining records were screened by referring to the full-text content. Studies presenting only correlation analyses without experimental validation, similar findings, or those concerning non-specific TFAP2 members were excluded. Moreover, review papers, non-English publications, no full-text availability, and preprint records were also excluded. Nine records were discarded for other reasons. Ultimately, 161 studies on the role of TFAP2 in cancer were included, while an additional ten references were incorporated to provide background information.
